# OnabotulinumtoxinA 155 U in medication overuse headache: a two years prospective study

**DOI:** 10.1186/s40064-015-1636-9

**Published:** 2015-12-30

**Authors:** Andrea Negro, Martina Curto, Luana Lionetto, Dorotea Crialesi, Paolo Martelletti

**Affiliations:** Department of Clinical and Molecular Medicine, Sapienza University, Rome, Italy; Regional Referral Headache Centre, Sant’Andrea Hospital, Rome, Italy; Department of Psychiatry, Harvard Medical School, Boston, MA USA; Bipolar and Psychotic Disorders Program, McLean Hospital, Belmont, MA USA; Advanced Molecular Diagnostics Unit, IDI Istituto Dermopatico dell’Immacolata-IRCSS, Rome, Italy; Molecular Medicine Department, Sant’Andrea Hospital, Sapienza University, Via di Grottarossa 1035-1039, Rome, Italy

**Keywords:** Chronic migraine, Medication overuse headache, Migraine abuse, Preventative therapy, OnabotulinumtoxinA

## Abstract

The efficacy and safety of OnabotulinumtoxinA 155-195 U (BOTOX^®^) in adults with chronic migraine (CM) were demonstrated in both the Phase III Research Evaluating Migraine Prophylaxis Therapy (PREEMPT) studies. However, data about its long-term efficacy and safety in clinical settings are scanty. Therefore, the objective of this study is to evaluate OnabotulinumtoxinA 155 U treatment in patients affected with CM and co-morbid medication overuse headache (MOH) over 2-year analysis. We prospectively evaluated 155 CM and MOH affected patients started on OnabotulinumtoxinA 155U (PREEMPT injection paradigm) between October 2010 and November 2011 and followed-up for 2 years. All patients failed to positively respond to previous multiple preventive therapies that were withdrawn before starting OnabotulinumtoxinA. Headache days, migraine days, acute pain medication intake days and Headache Impact Test (HIT)-6 score were used as efficacy measures, whereas safety was evaluated with side effects occurrence during the treatment phase. Baseline data were collected from patients headache diary referred to the previous month, and patients were evaluated every 3 months at the time of each injection. OnabotulinumtoxinA 155U significantly reduced the number of headache and migraine days (p < 0.001), acute pain medication intake days (p < 0.001) and HIT-6 score (p < 0.001) when compared with the baseline data. The reduction was significant after the first injection (p < 0.001), and gradually increased during the 2 years of treatment. Treatment related adverse events were transient and mild-moderate (e.g. headache, injection-site pain, eyelid ptosis, musculoskeletal weakness). This prospective 2-years analysis of efficacy and safety of long-term treatment with OnabotulinumtoxinA 155 U in patients affected with CM and MOH confirms the efficacy data from previous Randomized Clinical Trials for CM prophylaxis. Moreover, here we demonstrate that OnabotulinumtoxinA can be safely used for the long-term treatment of MOH comorbidity in CM.

**Electronic supplementary material:**

The online version of this article (doi:10.1186/s40064-015-1636-9) contains supplementary material, which is available to authorized users.

## Background

Migraine is the most frequent neurological disease observed in clinical practice. It is a primary headache disorder associated with an important socioeconomic burden (Steiner et al. 2011; Bloudek et al. 2012).

According to the results of the Global Burden of Disease Study 2013, headache disorders are the third cause of disability worldwide when considering the years of life lost because of disability, and migraine alone is ranked at the 6th place (GBDS 2015; Steiner et al. 2015).

Migraine can be episodic or chronic, depending on the frequency of the headache attacks. Compared to episodic migraine, Chronic Migraine (CM) is associated with higher disability, lower quality of life and greater health resource utilization. In fact, patients affected with CM often use multiple medications, both in prophylactic and in the acute treatment. Moreover, the excessive use of acute pain medications might increase the risk of medication overuse headache (MOH). CM has also been demonstrated to be an independent risk factor for cardiovascular disorders and ischemic stroke, and higher frequency of attacks might lead to a chronic intake of non-steroidal anti-inflammatory drugs (NSAIDs) and triptans, both associated with a greater likelihood of developing cardiovascular events (Tana et al. 2013). Therefore chronic migraine prevention and reduction of the attacks frequency might also be essential for the cardiovascular risk reduction.

The available drugs for the preventive treatment of CM belong to different classes (i.e. anti-epileptic, antidepressants, beta-blockers, calcium channel antagonists) and are generally used in mono-therapy. In fact, evidence about the preventive efficacy of drugs combinations is not sufficiently strong, and the efficacy of each single medication should be carefully evaluated alone. Moreover, the very frequent presence of comorbidities should drive the choice of a preventive migraine treatment, in order to reduce the patient’s medication intake and increase the clinical efficacy for both the disorders (Negro et al. 2010). Anyway, the response to a specific preventive drug is variable for each patient and not always predictable and fluctuates over time.

In 2010 the efficacy and safety of OnabotulinumtoxinA were evaluated in the prophylactic treatment of CM with a Randomized Clinical Trial, namely the “Phase III Research Evaluating Migraine Prophylaxis Therapy” (PREEMPT) clinical program. This consisted in two phases 3, multicenter double-blind, parallel-group, placebo-controlled studies (PREEMPT 1 and 2) (Aurora et al. 2010; Diener et al. 2010; Dodick et al. 2010). PREEMPT enrolled 1384 adult patients with CM in North America and Europe, which went through a 24-week randomized, double-blind, parallel-group, placebo-controlled phase followed by a 32-week open-label phase, with injections repeated every 12 weeks of 155-195 U of OnabotulinumtoxinA.

Our experience with OnabotulinumtoxinA for the prevention of chronic daily headaches started in 2001. At that time we administered injections of OnabotulinumtoxinA 100 U with an off-label use (Farinelli et al. 2006). The dose was later increased to 155 U in 2010 after the publication of the PREEMPT studies.

Since 2010, we administered OnabotulinumtoxinA 155 U injections in 31 sites every 3 months following the PREEMPT injection paradigm, without the administration of the additional 40 U.

In our Headache Clinic the preventive treatment with OnabotulinumtoxinA was offered to all the adults patients that were affected with CM with or without medication overuse (diagnosis made according with the IHS criteria 2004 revised in 2006) (Headache Classification Subcommittee 2004; Headache Classification Committee 2006). We offered such treatment only to patients with contraindications or lack of efficacy or tolerability to other preventive drugs (beta-blockers, calcium channel blockers, antiepileptics and antidepressants). We did not offer OnabotulinumtoxinA treatment to patients with co-morbid neuromuscular disorders, psychiatric diseases considered incompatible with such kind of treatment, pregnancy and breast-feeding.

OnabotulinumtoxinA was not approved by the Italian regulatory drug agency (AIFA) for the prevention of CM, therefore at that time we used it off-label and all patients had to give an explicit informed consent. All patients with criteria for MOH underwent withdrawal and detoxification therapeutic regimen before starting OnabotulinumtoxinA. A program of at least 3 cycles of injections was planned for every patient. At the time of the fourth injection the clinical response was evaluated and if there was a response (reduction ≥50 % of migraine days) the patient could continue the treatment, otherwise OnabotulinumtoxinA injections were discontinued.

With the except of the PREEEMPT studies, large and long term studies on OnabotulinumtoxinA efficacy and safety have not been published. Given our extensive clinical experience with OnabotulinumtoxinA, the aim of this study was to prospectively evaluate its long-term efficacy and safety in the treatment of CM and co-morbid MOH over 2-year period.

## Methods

The protocol was reviewed and approved by the Ethical Committee of Sant’Andrea Hospital, Sapienza University of Rome. Each patient gave a free, informed consent for the participation in the study and the analysis and publication of the protocol data.

We included in the study all the patients affected with CM with MOH that referred to our Headache Clinic between October 2010 and November 2011. We included only patients with criteria for MOH who had failed one or more withdrawal attempts, and all the CM patients who had received and failed other preventive therapies due to lack of efficacy or intolerable side effects. Any patient was allowed to take preventive oral medication during treatment with OnabotulinumtoxinA.

OnabotulinumtoxinA was administered in a Day Hospital setting and was properly charged to our National Health System. It was injected every 3 months (±1 week) following the PREEMPT injection paradigm: all patients received a dose of 155 U of OnabotulinumtoxinA administered to 31 injection sites across seven head and neck muscles using a “fixed-site, fixed-dose” injection paradigm (Blumenfeld et al. 2010). Up to 40 U of additional OnabotulinumtoxinA could have been administered at the physician’s discretion using a “follow the pain” strategy into 8 additional injection sites across three head and neck muscles (temporalis, occipitalis, and/or trapezius muscles) in the PREEMPT study (Blumenfeld et al. 2010), but we did not administer the additional dose.

Headache days, migraine days, and acute pain medication intake were used as efficacy measures. Baseline data were collected from patients headache diary referred to the previous month before starting OnabotulinumtoxinA, and patients were evaluated every 3 months at the time of each injection. Every 6 months patients were asked to fill the Headache Impact Test (HIT)-6, used as a measure of efficacy as well, and the results were compared with the baseline score. During the 24 months all adverse events (AEs), related to the drug, were registered and used as a safety measure.

### Statistical analysis

Continuous variables are reported as mean ± standard deviation, rates and categorical values are reported as subjects-counts and percentage. A paired *t* Test was used to compare the mean headache days, migraine days, medication intake days and HIT-6 score at baseline and at each cycle of injections after Hartley’s ƒ-Max test to ass equal variance of data. A Chi square test was used to compare categorical variables.

## Results

### Demographic and baseline headache characteristics

The initial included population was of 155 patients but only 132 patients completed the 2 years follow-up. Table 1 shows the reasons for OnabotulinumtoxinA discontinuation before 24 months.

**Table 1:** Reasons for OnabotulinumtoxinA discontinuation before 24 months

Total	N = 23 (14.8 %)
OnabotulinumtoxinA indepent AEs	5 (3.2 %)
Not effective	8 (5.2 %)
Pregnancy	2 (1.3 %)
Drop out	3 (1.8 %)
Personal reasons	5 (3.2 %)

*AEs* adverse events

Demographic and baseline headache characteristics of the 132 patients who completed the protocol are reported in Table 2.

**Table 2:** Baseline demographics and characteristics

	OnabotulinumtoxinA 155 U (n = 132)
Mean age, years	43.2 ± 13.5 (18–76)
Female, % (n)	81.8 (108)
Diagnosis of chronic migraine, years	7.6 ± 4.3 (0.5–10)
Mean years since onset of chronic migraine	10.2 ± 4.8 (1–40)
Headache days	22.3 ± 4.1
Migraine days	21.4 ± 4.3
Pain medication intake days	20.8 ± 4.5
HIT-6 score	68.9 ± 4.3
Patients with severe HIT-6 score, % (n)	93.9 (124)

Data are presented as mean ± standard deviation; *HIT* headache impact test, HIT-6 Scores of 36–49 indicate little or no impact; 50–55, some impact; 56–59, substantial impact; ≥60, severe impact

Baseline data refers to the previous month before starting OnabotulinumtoxinA

The majority of patients were female (81.8 %), with a mean age of 43.2 years (range 18–76). The average time from CM onset was 10.2 years (range 1–40 years). All patients overused acute pain medications during the previous month before starting OnabotulinumtoxinA treatment (baseline). Medication overuse was defined as simple analgesics intake for ≥15 days, or other medication classes or combination of multiple drug classes for ≥10 days, taken at least 2 days/week or more. The 93.9 % of patients had a severe (≥60) HIT-6 score (Table 2).

### Efficacy

**Fig. 1:**
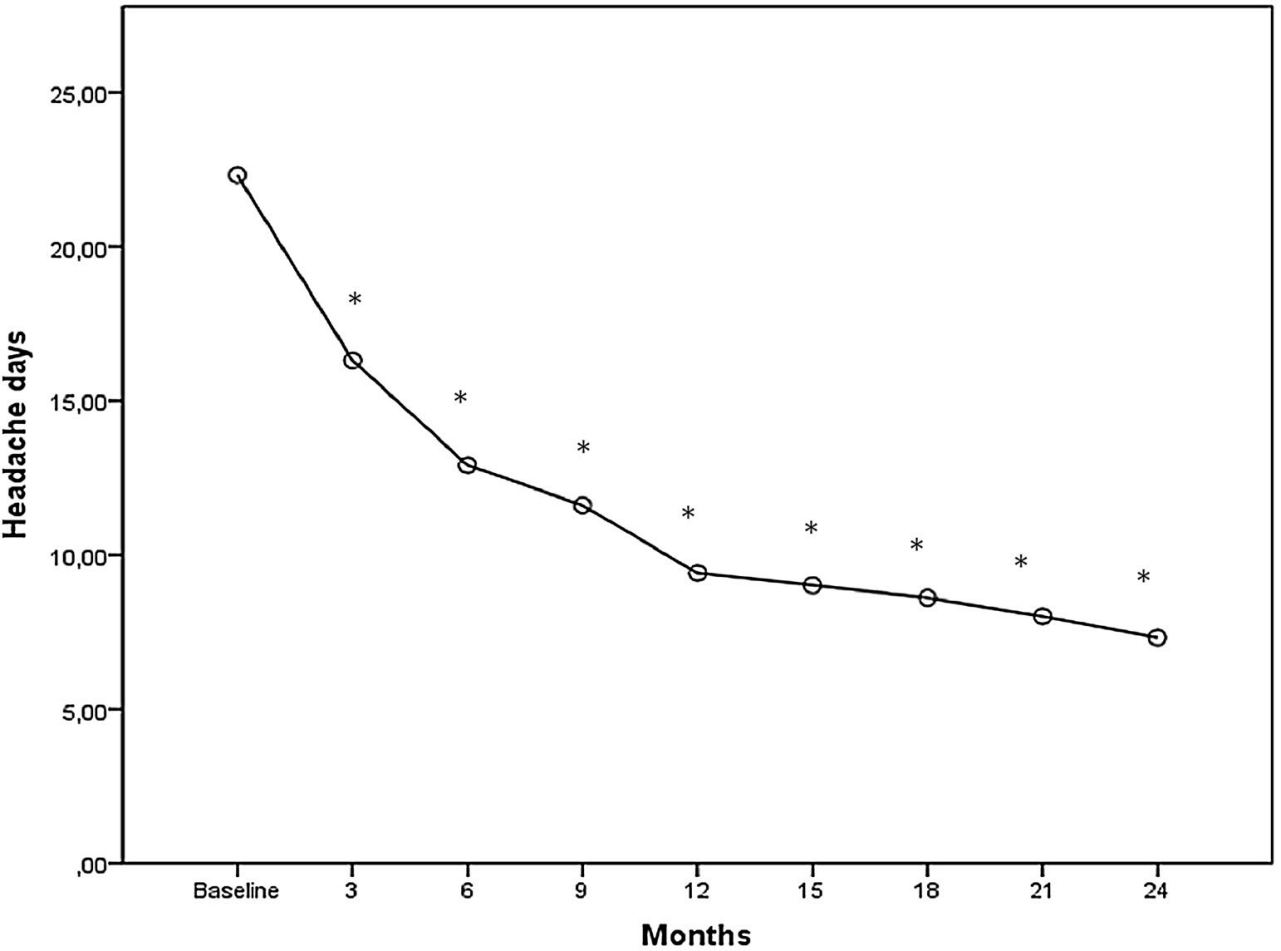
Mean change from baseline in frequency of headache days. *p < 0.001

**Fig. 2:**
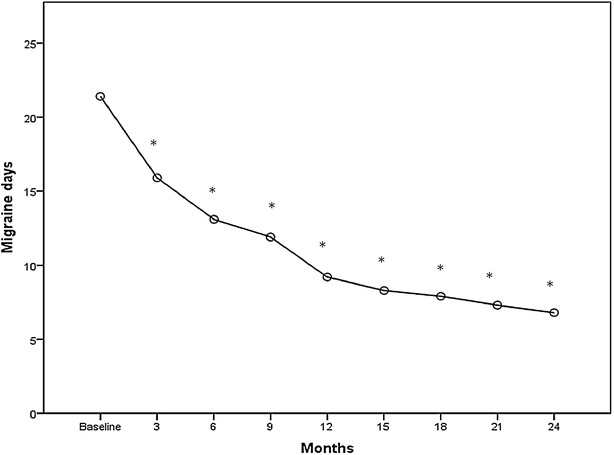
Mean change from baseline in frequency of migraine days. *p < 0.001

**Fig. 3:**
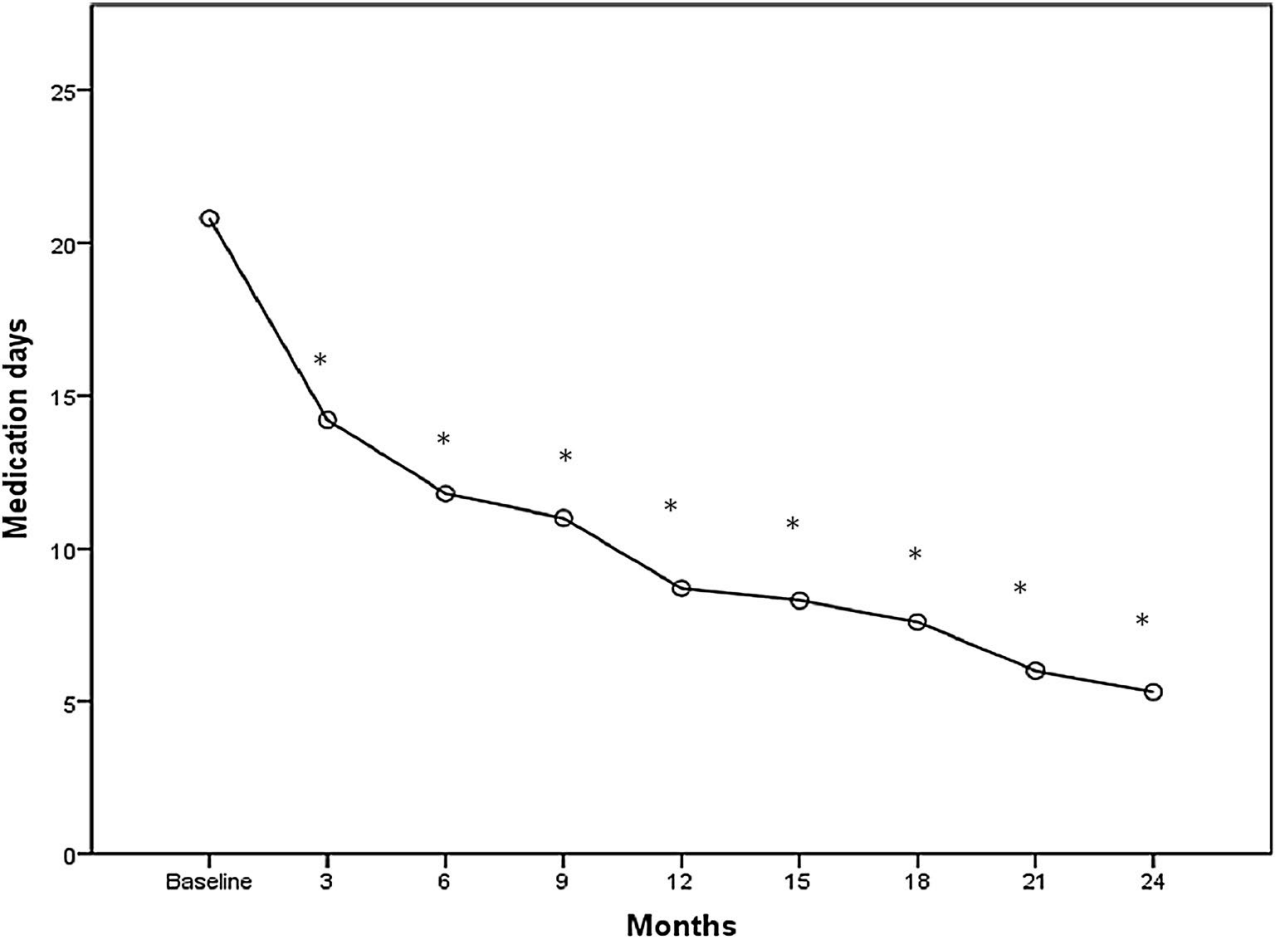
Mean change from baseline in monthly pain medication intake days. *p < 0.001

**Fig. 4:**
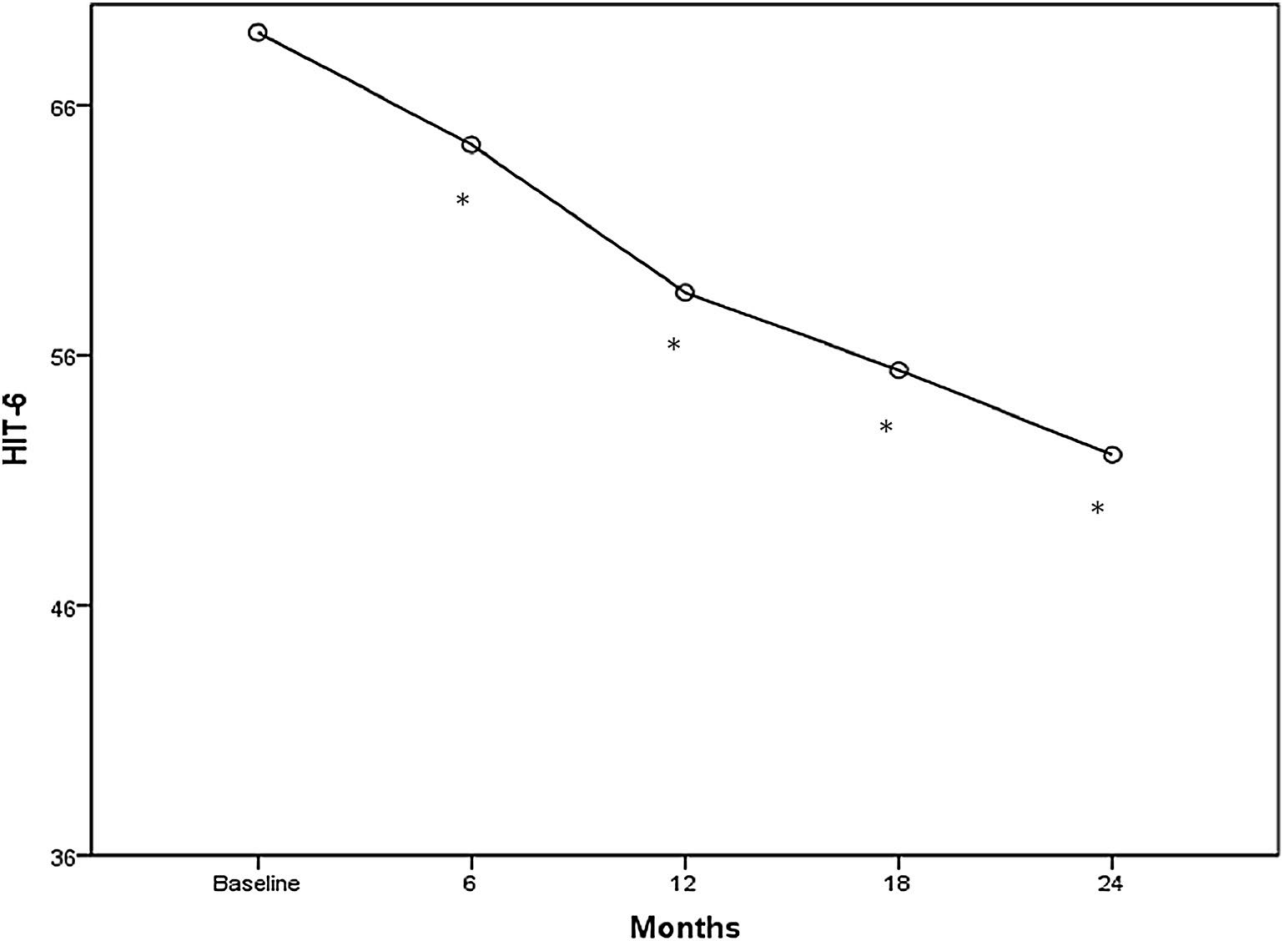
Mean change from baseline in total HIT-6 score. *p < 0.001

A comparison of all pre- and post-treatment outcomes is shown in Figs. 1, 2, 3, 4 and detailed data are given in Additional file 1: Tables S1–S5.

The headache days per month decreased significantly during the period of treatment from the first to the eighth session of therapy (pre 22.3 ± 4.1, post 7.3 ± 2.1; p < 0.001) (Fig. 1).

There was also a significant reduction of the migraine days per month during the period of treatment from the first to the eighth session of therapy (pre 21.4 ± 4.3, post 6.8 ± 2.3; p < 0.001) (Fig. 2).

Accordingly, also medication intake days decreased significantly during the period of treatment from the first to the eighth session of therapy (pre 20.8 ± 4.5, post 5.3 ± 1.7; p < 0.001) (Fig. 3).

The mean HIT-6 score decreased significantly during the period of treatment from the first to the last injection (pre 69.4 ± 4.9, post 52 ± 5.6; p < 0.001) (Fig. 4) and the proportion of patients with severe (≥60) HIT-6 score decreased as well (pre 93.9 %, post 22 %). After 2 years of treatment the mean HIT-6 score was ≤55 (some impact, little or no impact) in 68.2 % of patients and ≤49 (little or no impact) in 40.9 % of patients (data not shown). Notably, all the efficacy measures showed a progressive decline over the 2-year follow-up, with significant reductions at each time point calculated only versus baseline measures. Notably, after the 4th injection (12 months), the extent of decreases of the mean headache, migraine and pain medication intake days appears to be less prominent than within the first 12 months of treatment.

### Safety and tolerability

No severe treatment-related AEs were observed (Table 3). Severity of the AEs was mild to moderate and was not a reason to interrupt OnabotulinumtoxinA injections in any case. All the AEs lasted for less than a week (e.g. headache, injection-site pain) to a maximum of 2 months (e.g. eyelid ptosis, musculoskeletal weakness).

**Table 3:** Summary of the overall treatment-related AEs reported in 24 months

Adverse event observed	Number of patients/132 (%)
Total treatment-related AEs	23 (17.5)
Injection-site pain	4 (3.3)
Neck pain	5 (3.8)
Musculoskeletal weakness	5 (3.8)
Eyelid ptosis	4 (2.9)
Headache	5 (3.7)

*AEs* adverse events

Neck pain and musculoskeletal stiffness occurred in >75 % of the cases during the first three cycles of injections. Conversely, other AEs, as eyelid ptosis, injection-site pain and headache did not show any correlation with the treatment cycle (data not shown).

## Discussion

For decades several classes of drugs have been used for the prevention of primary chronic headaches showing different levels of efficacy and safety. Considering the large number of molecules available for each class and their association with severe class-related contraindication, it is not surprising to note that there are no published data comparing safety and efficacy between all the classes. It is worth it to remember that none of these drugs are currently approved for CM prophylaxis.

In 2010, on the basis of the results of PREEMPT clinical program, the Medicines and Healthcare Products Regulatory Agency (MHRA) in the UK, and later the Federal Drug Administration (FDA) in USA, approved OnabotulinumtoxinA injection therapy for the prevention of headaches in adults with CM. In 2011 also the European Medical Agency (EMA) approved Botox^®^ for treatment of CM. OnabotulinumtoxinA was the first and, so far, the only prophylactic treatment to receive a specific license for patients with CM. Recently the European Headache Federation proposed a consensus statement for a new definition of refractory CM (Martelletti et al. 2014) which includes also the OnabotulinumtoxinA treatment failure as a diagnostic criteria for the definition of refractory CM.

A comparison between our results and the PREEMPT pooled data might be helpful to better understand OnabotulinumtoxinA long-term safety and efficacy (Dodick et al. 2010).

The majority of baseline characteristics of our sample of patients were similar to the PREEMPT population: mean headache days (22.3 vs. 19.9), mean migraine days (21.4 vs. 19.1), mean HIT-6 score (69.4 vs. 65.5) and percent with severe (≥60) HIT-6 score (93.9 vs. 93.5).

At the same time, our population had some important differences. All our patients had MOH (vs. 64.8 %) and had previously failed one or more prophylaxis medications (38 % in PREMPT had never received a preventive treatment). Therefore, we might assume that our sample of patients had a more severe CM. Nevertheless, we report a similar OnabotulinumtoxinA efficacy compared to PREEMPT study, at least in the first year of treatment (the PREEMPT study considered only a 1 year follow-up).

To date, similar efficacy is indicated by the similar outcome measures reduction at PREEMPT 24 weeks and our 6 months: mean headache days reduction (−8.4 vs. −9.4), mean migraine days reduction (−8.2 vs. −8.4), mean acute pain medication intake reduction (−10.1 vs. −9), mean HIT-6 score reduction (−4.8 vs. −5.1), proportion of patients with severe (≥60) HIT-6 score (67.6 vs. 77.3 %). Moreover, we can also compare outcome measures reduction at PREEMPT 56 weeks and our at 15 months: mean headache days frequency reduction (−11.7 vs. −13.2), mean migraine days frequency reduction (−11.2 vs. −13.1), mean reduction in acute pain medication intakes (−15.4 vs. −12.6), mean HIT-6 score (−7.7 vs. −10.9), proportion of patients with severe (≥60) HIT-6 score (50.6 vs. 44.7 %).

The safety and tolerability profile that emerged from our study seems to be better than that of pooled data from PREEMPT (total treatment-related AEs: 17.5 vs. 29.4 %).

In our Headache Clinic, the rate of patients discontinuing the therapy for AEs related to OnabotulinumtoxinA was around the 3 %, mostly represented by women that experienced neck muscle weakness and eyelid ptosis.

In other real-life studies, in order to prevent the re-occurrence of the local toxin AEs the interested injection sites were avoided or the dose was incorrectly reduced to the half (Cernuda-Morollón et al. 2015). We do not feel to recommend this kind of behavior since most of the local AEs can be successfully avoided by improving the injection practice. Moreover, the preventive effect of OnabotulinumtoxinA seems to be dose dependent, therefore avoiding sites of injections or reducing the dose per site can be counterproductive.

The overall OnabotulinumtoxinA efficacy in reducing headache and migraine days, and therefore the pain medication use and overuse without any relevant and severe side effect is of central importance also for reducing CM associated disability, increasing patients quality of life and reducing health resource utilization. Furthermore, reducing the attacks frequency and the pain medication use and abuse might be also an essential part of the cardiovascular risk reduction in patients affected with CM (Tana et al. 2013).

A particularly interesting finding of this study is the progressive improvement in all measures during the 2 years of follow-up. Although the extent of the headache, migraine and medication intake days reduction tends to be less prominent after the 4th injection, this data demonstrate that OnabotulinumtoxinA efficacy is at least maintained and possibly continues to improve over time.

The results of other studies in a real-life setting have been recently published and confirm the efficacy and safety of OnabotulinumtoxinA, even if differences in some important features between the studies should be underlined. Pedraza et al. (2015) evaluated fifty-two patients treated with one injection of 155 U, with only 39 receiving a second treatment; 82.7 % of them presented MOH, and the use of preventive therapy during the study was allowed. Grazzi and Usai (2015) evaluated forty-six patients treated with 3 injections sessions and 20 patients treated with 5 injections; all of them received the dosage of 155 U for each session and they were all suffering from CM with MOH. Notably, it was not mentioned if other preventive therapies were allowed.

Khalil et al. (2014) showed that OnabotulinumtoxinA 155 U reduced the number of headache and migraine days in a prospective study of 254 adults with CM, and increased the number of headache-free days only for the first month after treatment. Cernuda-Morollón et al. (2015) reported their experience in a sample of 132 CM patients treated with OnabotulinumtoxinA 155–195 U following the PREEMPT protocol. They showed a long-term efficacy to OnabotulinumtoxinA injections after 1 year in about three quarters of the patients. The same efficacy was maintained in all the 20 patients that were still treated after 4 years. However, in the paper there is no mention about the dosage (155 or 195 U).

Our study has two major points of strength. First, the same physician that had received a proper training as injector was the same that followed each patient up over the 2 years. This might have contributed in reducing the variation in the injection sites, possibly linked to the different operators performing the ptotocol. Therefore, the importance of a proper training offered to each physician approaching to this injective therapy might be central for OnabotulinumtoxinA treatment (Blumenfeld et al. 2010). Second, we evaluated a single dosage (155 U) in a large population for a relatively long period (8 treatments in 2 years).

Limitations of our study are also noteworthy. The first limitation is the absence of a control group; the second is the lack of collected descriptive data for some of the outcomes (e.g. headache and migraine episodes, moderate/severe headache days, Migraine-Specific Quality of Life questionnaire). The choice to not collect excessive descriptive data has been made to increase the compliance of our patients in fulfilling the diaries. In fact, usually patients report in diaries only information related to headache days, migraine days, day of utilization of drug and any adverse event. Similarly, the primary end point of the pooled data of PREEMPT was the frequency of headache days, which is considered more sensitive than headache episodes and fulfills recently proposed clinical trial guidelines for evaluating headache prophylaxis in CM (Silberstein et al. 2008). Another limitation is the absence of data about the length of time of effectiveness of treatment within the 3 months: in fact, we calculated the headache days, migraine days, and medication intake days as a mean of the overall days reported by the patients in the previous 3 months, without considering single months or weeks as outcome measures. This implies that patients experiencing variations in the OnabotulinumtoxinA length of action within the 3 months might have been not recognized.

The last criticism about this study could be represented by the selection of the population. We decided to prospectively treat and evaluate only patients with CM and coexisting MOH, which precluded the diagnosis of CM according to the ICHD-II (Headache Classification Subcommittee 2004; Headache Classification Committee 2006). The PREEMPT studies received the same critics. MOH constitutes a plus of CM and it is hard to think about its appearance not being related to CM itself, unless patients attempt counterproductive stoicism. Since MOH does not stand alone, it is our opinion that it should be at least considered a complication of CM and not just a simple form of secondary headache (Negro and Martelletti 2011). Accordingly prospective data in real-life clinical practice from the Hull Migraine Clinic shows no difference in the therapeutic outcome in patients with or without analgesic overuse (Ahmed et al. 2015).

## Conclusions

Our data from a real-life setting confirm OnabotulinumtoxinA efficacy and safety evidence from previous RCT studies.

OnabotulinumtoxinA proved to have indisputable advantages over other drugs used for decades for CM prophilaxys: higher efficacy and tolerability, that are maintained even after a long period of treatment. In our opinion, OnabotulinumtoxinA should be considered the gold standard preventive therapy for CM.

## Additional file

10.1186/s40064-015-1636-9 Supplementary Tables 1–5.
