# Supplementary material for: OnabotulinumtoxinA 155 U in medication overuse headache: a two years prospective study
Source: Springerplus. 2015 Dec 30;4:826. doi: 10.1186/s40064-015-1636-9 (PMC4695480; doi:10.1186/s40064-015-1636-9)
Supplement: Supplementary file 1 — 10.1186/s40064-015-1636-9 Supplementary Tables 1–5. [file 40064_2015_1636_MOESM1_ESM.docx]

**Supplementary Material**

**Table S1. Mean change from baseline in frequency of headache days.**

| Table S1. Mean change from baseline in frequency of headache days | | | |
| --- | --- | --- | --- |
|  | **OnabotulinumtoxinA 155 U (n=132)** | **t** | ***P* value** |
| Baseline (1^st^ injection) | 22.3 ± 4.1 | -- | -- |
| 3 months (2^nd^ injection) | 16.3 ± 2.7 | 25.9 | <0.001 |
| 6 months (3^rd^ injection) | 12.9 ± 2.6 | 34.7 | <0.001 |
| 9 months (4^th^ injection) | 11.6 ± 2.2 | 35.6 | <0.001 |
| 12 months (5^th^ injection) | 9.4 ± 2.9 | 67.1 | <0.001 |
| 15 months (6^th^ injection) | 9.0 ± 2.8 | 62.9 | <0.001 |
| 18 months (7^th^ injection) | 8.6 ± 2.6 | 62.3 | <0.001 |
| 21 months (8^th^ injection) | 8.0 ± 2.3 | 60.2 | <0.001 |
| 24 months | 7.3 ± 2.1 | 60.1 | <0.001 |
| Data are presented as mean ± standard deviation | | | |

**Table S2. Mean change from baseline in frequency of migraine days.**

| Table S2. Mean change from baseline in frequency of migraine days | | | |
| --- | --- | --- | --- |
|  | **OnabotulinumtoxinA 155 U (n=132)** | **t** | ***P* value** |
| Baseline (1^st^ injection) | 21.4 ± 4.3 | -- | -- |
| 3 months (2^nd^ injection) | 15.9 ± 2.8 | 20.7 | <0.001 |
| 6 months (3^rd^ injection) | 12.4 ± 2.5 | 27.7 | <0.001 |
| 9 months (4^th^ injection) | 11.3 ± 2.3 | 29.0 | <0.001 |
| 12 months (5^th^ injection) | 9.2 ± 2.8 | 57.3 | <0.001 |
| 15 months (6^th^ injection) | 8.3 ± 3.0 | 56.8 | <0.001 |
| 18 months (7^th^ injection) | 7.9 ± 3.0 | 60.7 | <0.001 |
| 21 months (8^th^ injection) | 7.3 ± 2.7 | 60.5 | <0.001 |
| 24 months | 6.8 ± 2.3 | 54.8 | <0.001 |
| Data are presented as mean ± standard deviation | | | |

**Table S3. Mean change from baseline in monthly pain medication intake days.**

| Table S3. Mean change from baseline in monthly pain medication intake days | | | |
| --- | --- | --- | --- |
|  | **OnabotulinumtoxinA 155 U (n=132)** | **t** | ***P* value** |
| Baseline (1^st^ injection) | 20.8 ± 4.5 | -- | -- |
| 3 months (2^nd^ injection) | 14.2 ± 2.8 | 24.0 | <0.001 |
| 6 months (3^rd^ injection) | 11.8 ± 2.4 | 26.1 | 0.798 |
| 9 months (4^th^ injection) | 11 ± 2.1 | 28.4 | <0.001 |
| 12 months (5^th^ injection) | 8.7 ± 2.7 | 48.2 | <0.001 |
| 15 months (6^th^ injection) | 8.3 ± 3.0 | 49.1 | <0.001 |
| 18 months (7^th^ injection) | 7.6 ± 2.9 | 50.5 | <0.001 |
| 21 months (8^th^ injection) | 6.0 ± 2.3 | 49.9 | <0.001 |
| 24 months | 5.3 ± 1.7 | 47.2 | <0.001 |
| Data are presented as mean ± standard deviation | | | |

**Table S4. Mean change from baseline in total HIT-6 score.**

| Table S4. Mean change from baseline in total HIT-6 score | | | |
| --- | --- | --- | --- |
|  | **OnabotulinumtoxinA 155 U (n=132)** | **t** | ***P* value** |
| Baseline (1^st^ injection) | 68.9 ± 4.3 | -- | -- |
| 6 months (3^rd^ injection) | 64.4 ± 5.0 | 22.2 | <0.001 |
| 12 months (5^th^ injection) | 58.5 ± 3.7 | 41.0 | <0.001 |
| 18 months (7^th^ injection) | 55.4 ± 4.9 | 42.9 | <0.001 |
| 24 months | 52.0 ± 5.6 | 46.6 | <0.001 |
| Data are presented as mean ± standard deviation;HIT, Headache Impact Test | | | |

| Table S5. Change from baseline in percentage of patients with severe impact (HIT-6 score ≥60) | |
| --- | --- |
|  | **OnabotulinumtoxinA 155 U (n=132)**  **% (n)** |
| Baseline (1^st^ injection) | 93.9 (124) |
| 6 months (3^rd^ injection) | 77.3 (102) |
| 12 months (5^th^ injection) | 44.7 (59) |
| 18 months (7^th^ injection) | 37.1 (49) |
| 24 months | 22 (29) |
| HIT, Headache Impact Test | |

**Table S5. Change from baseline in percentage of patients with severe impact (HIT-6 score ≥60).**
